# Supplementary material for: A Pacific Oyster-Derived Antioxidant, DHMBA, Protects Renal Tubular HK-2 Cells against Oxidative Stress via Reduction of Mitochondrial ROS Production and Fragmentation
Source: Int J Mol Sci. 2023 Jun 13;24(12):10061. doi: 10.3390/ijms241210061 (PMC10298743; doi:10.3390/ijms241210061)
Supplement: Supplementary file 1 [file ijms-24-10061-s001.zip › ijms-2424764-supplementary.pdf]

Table S1 List of primers for qRT-PCR

gene name	primer	Accession No.
mitochondrial-dependent apoptosis-related genes		
BIK	F: 5'- GACCTGGACCCTATGGAGGAC -3'	NM_001197.5
	R: 5'- CCTCAGTCTGGTCGTAGATGA -3'	
ENDOG	F: 5'- AGCAGGTGGGCAAATTGAG -3'	NM_004435.2
	R: 5'- CCAGGATGTTTGGCACAAAGAG -3'	
mitochondrial fission and fusion-related genes		
OPA1	F: 5'- GCTGAACGCAGTATTGTTACAG -3'	NM_130835.3
	R: 5'- TATAGCTTCAATGCTTTCAGAGCT -3'	
DRP1	F: 5'- GAGTTACTGGTGAAGCGGCA -3'	NM_001278465.2
	R: 5'- ATTGCCACTAAGTTATGGACCA -3'	
FIS1	F: 5'- AAGAGCACGCAGTTTGAGTAC -3'	NM_016068.3
	R: 5'- CTGGGGCTCTGTCTGCAGC -3'	
mitochondrial biosynthesis-related genes		
PGC1α	F: 5'- TGAACTGAGGGACAGTGATTTC -3'	NM_001330753.2
	R: 5'- CCCAAGGGTAGCTCAGTTTATC -3'	
NRF1	F: 5'- GTATCTCACCCTCCAAACCTAAC -3'	NM_001040110.2
	R: 5'- CCAGGATCATGCTCTTGTACTT -3'	
TFAM	F: 5'- ATAGGCACAGGAAACCAGTTAG -3'	NM_001270782.2
	R: 5'- GCAGAAGTCCATGAGCTGAATA -3'	

autophagy-related genes		
LC3B	F: 5'- ACCATGCCGTCGGAGAAGAC -3'	NM_001085481.3
	R: 5'- TCTCGAATAAGTCGGACATCTTCTACTCT -3'	
GABARAPL1	F: 5'- ATCGGAAAAAGGAAGGAGAAAAGATC -3'	NM_001363598.2
	R: 5'- CAGGCACCCTGGCTTTTGG -3'	
BNIP3	F: 5'- AGCGCCCGGGATGCA -3'	NM_004052.4
	R: 5'- CCCGTTCCCATTATTGCTGAA -3'	
BNIP3L	F: 5'- CTGCGAGGAAAATGAGCAGTCTCT -3'	NM_004331.3
	R: 5'- GCCCCCCATTTTTCCCATTG -3'	
FUNDC1	F: 5'- GAAACGAGCGAACAAAGCAG -3'	NM_173794.4
	R: 5'- GCAAAAAGCCTCCCACAAAT -3'	
PINK1	F: 5'- CGTTGGACACGAGACGCTTGCA -3'	NM_032409.3
	R: 5'- GCAATGTAGGCATGGTGGCTTCATAC -3'	
OPTN	F: 5'- AAGGAGCAACTGGCATTGCA -3'	NM_001008212.2
	R: 5'- TCTCCATCAAGGACTGCCTG -3'	
CALCOCO2	F: 5'- TCTTTGCCGTATCAAGTACCTACT -3'	NM_001261393.2
	R: 5'- GGCATTTCTTGATGGAGAGCG -3'	
house keeping gene		
β-actin	F: 5'- ATAGCACAGCCTGGATAGCAACGTAC -3'	NM_001101.5
	R: 5'- CACCTTCTACAATGAGCTGCGTGTG -3'	

F: Forward; R: Reverse.
